# Comparative Analysis of Plastic Waste Management Options Sustainability Profiles

**DOI:** 10.3390/polym17152117

**Published:** 2025-07-31

**Authors:** Madalina-Maria Enache, Daniela Gavrilescu, Carmen Teodosiu

**Affiliations:** Department of Environmental Engineering and Management, “Cristofor Simionescu” Faculty of Chemical Engineering and Environmental Protection, “Gheorghe Asachi” Technical University of Iasi, 73 D. Mangeron Street, 700050 Iasi, Romania; madalina-maria.enache-cozma@student.tuiasi.ro

**Keywords:** plastic waste, sustainability assessment, end-of-life treatment, waste infrastructure, US EPA WARM

## Abstract

Efficient plastic waste end-of-life management is a serious worldwide environmental issue motivated by growing waste production and negative effects of wrongful disposal. This study presents a comparative overview of plastic waste management regimes within the European Union (EU), the United States of America (USA), and Romania, ranked with circular economy goals. By using the United States Environmental Protection Agency (US EPA) Waste Reduction Model (WARM), version 16, the study provides a quantified score to greenhouse gas (GHG) emissions within three large options of management: recycling, energy recovery through combustion, and landfilling. The model setup utilizes region-specific information on legislation, base technology, and recycling efficiency. The outcomes show that recycling always entails net GHG emissions reductions, i.e., −4.49 kg CO_2e_/capita/year for EU plastic waste and −20 kg CO_2e_/capita/year for USA plastic waste. Combustion and landfilling have positive net emissions from 1.76 to 14.24 kg CO_2e_/capita/year. Economic indicators derived from the model also show significant variation: salaries for PET management amounted to USD 2.87 billion in the EU and USD 377 million in the USA, and tax collection was USD 506 million and USD 2.01 billion, respectively. The conclusions highlight the wider environmental and socioeconomic benefits of recycling and reinforce its status as a cornerstone of circular-economy sustainable plastic waste management and a strategic element of national development agendas, with special reference to Romania’s national agenda.

## 1. Introduction

The worldwide management of plastic waste, which includes PET (polyethylene terephthalate), HDPE (high-density polyethylene), LDPE (low-density polyethylene), PP (polypropylene), and PE (polyethylene), create complex sustainability challenges. The problems encompass environmental contamination and excessive waste generation and toxic properties of plastics. According to recent data, dangerous chemical substances that leach from plastic products penetrate the environment, thus threatening human health and ecosystems [1]. All polymer types are marked with a recycling number (code) between 1 and 7, and they differ in chemical and physical characteristics. Some are heavily recycled, while others involve challenging processes or are recalcitrant to recycling. For PET, HDPE, and PP material processing, the primary method is mechanical recycling, i.e., operations including sorting, cleaning, pelletizing into granules, and reuse as raw material. Conversely, plastics such as PET, PP, polyvinyl chloride (PVC) and polystyrene (PS) are chemically recyclable, where the waste plastics are converted to monomers or fuels by sophisticated technologies and, in the case of PVC, with one extra step of dechlorination. For non-recyclable or mixed waste, energy recovery by incineration is used, which allows receiving some energy but is extremely detrimental to the environment. Finally, non-recyclable parts end up in landfills, causing significant damage to nature. Contemporary issues include the requirement of adequate waste separation, an important factor in the improvement of recycling efficiency. There is the possibility of using automated machinery, for instance, artificial intelligence (deep learning)-based equipment to immediately and precisely determine plastic types. Additionally, recycling of PET and HDPE is most optimal and extensive, whereas PVC and PS marked with code 7 remain challenging to recycle, needing special treatments and technologies [2,3,4]. Microplastics have emerged as a major environmental issue because they negatively impact both soil and water environments. The absorption properties of various pollutants by microplastics determine their availability in ecosystems [3]. Research has shown that HDPE and PET plastics act as supplementary metal sources, which makes environmental contamination more complex [4]. The waste processing methods of pyrolysis and hydrothermal liquefaction demonstrate potential to transform waste into valuable materials. The approaches for plastic waste reduction and recycling improvement come with substantial expenses and operational difficulties [5]. Table 1 presents the main polymer categories and their codes.

To tackle all these problems, numerous recycling methods have been patented to improve the quality of plastics that are recycled. One of these innovative methods is plastic waste being used in making concrete so that products are made with low thermal conductivity and less plastic environmental burden. Second, there exist sophisticated sorting technologies based on near-infrared (NIR) spectroscopy that are vital in increasing recycling efficiency. Notwithstanding these developments, recycling technologies are still beset with important challenges, most notably contamination with nonolefin polymers. Contamination by such polymers has an adverse impact on the mechanical performance of recycled plastic panels, rendering the entire recycling process complicated [13,14]. Assessing the sustainability of polymers requires careful observation of all polymer classes and avoiding the use of mixed plastic fractions. By isolating and testing each polymer individually, their composition, properties, and recyclability can be ascertained with accuracy, and better-informed choices and more efficient recycling techniques can be used [14]. Table 2 presents the advantages of individual polymer analysis vs. mixed fractions.

The increasing volume of global waste, particularly mismanaged plastic waste (MPW), places significant pressures on waste management systems worldwide. Driven by a linear consumption model centered on plastic, this issue leads to environmental contamination across all ecosystems. All regions of the world are struggling to some extent with poor plastic waste management. In Africa, a large proportion of plastic waste arrives legally or illegally on the continent, and recycling rates are very low. Collection and recycling infrastructure is insufficient, leading to high rates of poor waste management [16]. Asia is known to have a disproportionately high burden of poorly managed plastic waste [17]. In Europe, the Mediterranean and Black Sea regions generate 90% of poorly managed plastic waste from coastal areas, which contributes significantly to marine pollution. Even though many European Union (EU) countries have improved waste management, the increase in the total volume of waste and exports to countries with poor infrastructure may exacerbate the problem [18]. The United States (US) faces significant challenges in managing plastic waste, despite its advanced infrastructure. In 2019, approximately 86% of plastic waste in the US was landfilled, 9% was incinerated, and only 5% was recycled. This represents a significant economic and energy loss. Much of the plastic waste is not recycled, and a significant proportion ends up in the environment or being exported to countries with weaker management systems [19].

Romania faces similar challenges due to inefficient waste management practices and high plastic consumption, which ultimately pollutes the environment. According to the Romanian Court of Accounts, the country remains largely reliant on landfills, while public resistance to circular economy measures—such as repairing products, reducing single-use plastics, and implementing separate waste collection—hinders progress. Additional obstacles include illegal plastic waste trade, institutional challenges in policy development, market restrictions on recycled materials, and limited corporate engagement in sustainability initiatives. As a result, Romania ranks among the EU countries with the poorest performance in waste management, particularly in terms of waste generation relative to gross domestic product (GDP), waste treatment efficiency, and the integration of recycled materials into the economy [20].

According to data provided by the Romanian Environmental Guard, a study carried out with results on macro- and microplastic pollution until 2020 concluded that the supervision of the municipal waste management sector revealed that urban and rural municipalities, in addition to the business sector, are still linked to environmental faults regarding the failure to comply with the separation at the source of municipal waste, leading to poor waste traceability with significant repercussions on the low recycling rates of plastic waste and exposure to illegal waste trade activities. These poor results in terms of waste quantities collected were attributed to, in addition to the low levels of coverage with efficient collection services, the low involvement of citizens, organizations, and institutions in the process. Essentially, an increased level of shared knowledge is needed, including through educational curricula at all levels. Practically, the current culture of “random dumping” that fuels plastic pollution in the natural environment should be eliminated [21]. Unsafe disposal practices that could generate plastic pollution of the natural environment, such as random/illegal dumping, littering, open burning of plastics, and disposal in noncompliant landfills, lead to poor management of plastic waste. These practices in Romania are linked to poor logistics, especially in rural areas. The Circular Economy Strategy highlighted the following existential problems for plastic recycling: poor management of plastic waste, lack of data and research, slow implementation of circular economy principles, and barriers of mentality and infrastructure. The closure of noncompliant landfills is followed by a post monitoring process to detect environmental pollution risks [21]. Although Romania recently implemented the deposit return scheme (DRS), a significant step forward contributing to a visible increase in the recycling rate of PET bottles, the situation remains complex for other polymer fractions. The initiative demonstrates the potential of well-structured mechanisms to positively influence consumers’ behavior and effectively route plastic waste to recycling processes. The figures for April 2025 are eloquent in this respect: of the 272,552,624 pieces (equivalent to 9,342,639 kg) of DRS plastic packaging placed on the market, 205,369,318 pieces (or 7,098,901 kg) were returned, resulting in an impressive 75.35% return rate for plastics [22]. However, at a national level, the lack of a consolidated and publicized recycling rate for all polymer fractions (such as HDPE, PVC, LDPE, PP, PS, and other composite materials) is a major gap. Without a clear picture of the performance of each type of plastic, it is difficult to identify inefficiencies, plan the necessary infrastructure, and implement truly sustainable and scalable end-of-life treatment solutions for all the plastic waste produced. Therefore, while the DRS is a success story for PET, the challenge remains to extend its efficiency to the entire spectrum of plastic waste to ensure sustainable and comprehensive management [21].

Plastics waste management varies substantially across regions of Europe, the United States of America (USA), and Asia, with conditional differences based on the degree of regulation, infrastructure, public awareness, and technological progress [22]. The European Union (EU) is a leading player in setting recycling policies, with its commitments on transition to a circular economy aiming to promote that by 2030, all plastic packaging should be reusable or recyclable. However, only 30% of plastic waste is recycled; the rest is burned (39%) or landfilled (31%). Furthermore, a large volume of recycled materials is shipped to Asia, creating environmental concerns [23,24]. The USA faces a long-standing reliance on landfilling despite recent advances in mechanical recycling and energy recovery. Environmental regulations are weaker than in the EU, but there is a high level of interest in sustainable alternatives and reducing uncontrolled disposal. Asia produces large masses of plastic waste with flexible and often inadequate disposal methods. China banned imports of plastic waste in 2017, producing a redirection of the flow to other Asian countries, which have started to adopt stricter measures. Insufficient waste management is starting to pollute the oceans and freshwater ecosystems. Efficient strategies require a regionalized approach, equally prioritizing chemistry, technological innovations, and strict regulations, e.g., chemical and biological recycling, the use of artificial intelligence in automated sorting, and the empowerment of producers to apply the principle of extended responsibility. Linked to these are public involvement and educational campaigns to promote zero-waste culture and change collective behavior [25,26,27].

The attraction of this approach to the perception of truth means a nuanced examination of the actors of the respective assumption. From this point of view, Romania, as an EU member state, is a relevant case. Its incorporation into the discussion not only allows an analysis of the way in which European directives are transposed at the national level but reveals the complexity of transition to a circular economy in a country with a distinct socioeconomic and infrastructural specificity. The representation of Romanian reality brings a valuable contrast and completes the global view, indicating on one hand the truly relevant results and on the other the challenges that remain in the exploitation of plastic waste recycling and treatment strategies. With respect to major barriers, poor management of waste collection and treatment facilities is a major barrier, particularly in low- and middle-income countries, where wastage disposal is not sufficiently treated in more than 90% of cases. Within normative space, there is limited harmonization of regulation of toxic plastic waste management. Furthermore, illicit export of toxic wastes from industrialized nations to developing nations with unsound regulations is perpetuating the issue across the globe [28]. The main objectives of this study are: (a) to comparatively assess the efficiency and impact of different end-of-life treatment strategies for plastic waste in the European Union and United States of America, with an in-depth analysis of the Romanian context and its progress towards a circular economy; (b) to compare the rates and methods of recycling plastic waste between EU and US, highlighting the main differences and strategic approaches; (c) to analyze the various end-of-life treatment options for plastic waste in EU and US, determining their environmental impact as quantified by the United States Environmental Protection Agency Waste Reduction Model (US EPA WARM); (d) to assess the current state of plastic waste recycling and management in Romania, identifying both progress in implementing circular economy principles and persistent challenges; and (e) to identify good practices from the European Union and United States that could be adopted in Romania and to highlight the gaps in the national plastic waste management system.

The novelty of our study is to provide a comparison among the European Union, United States of America, and Romania with the support of the US EPA WARM methodology, considering differences in environmental policies, infrastructures/technologies, recycling rates, and economic and practical considerations, such a comparison not having been elaborated so far.

## 2. Methodology

### 2.1. Waste Reduction Model (WARM)

The United States Environmental Protection Agency (US EPA) Waste Reduction Model (WARM), version 16, is an educational tool designed to support public and private organizations, local authorities, and stakeholders in making decisions about waste management options. It provides estimations of greenhouse gas (GHG) reductions, energy savings, and economic impacts of various waste management options, such as recycling, composting, anaerobic digestion, incineration, and landfilling. The WARM supports the assessment of management options and helps in making sustainable decisions in resources management in the context of climate change. The WARM can be used to analyze emissions, energy consumption, and economic factors for various materials commonly found in municipal solid waste and construction and demolition waste, using the following units of measurement: metric kg of CO_2_ equivalent (Kg CO_2e_), energy units (million BTU), labor hours, wages (USD), and taxes (USD). The current version of WARM, version 16, released in December 2023, contains 61 materials and products, divided into two main categories: Municipal Solid Waste (MSW) and Construction and Demolition Waste (C&D). Each material is accompanied in WARM by detailed emission factors, which include information on production from recycled and virgin materials, energy requirements, and end-of-life management. The WARM analyzes baseline scenarios (current practices) against alternative methods, such as recycling, incineration, and landfilling, to assess the effects of GHG emissions, energy effects, economic effects through wages and taxes, and social effects through the number of hours of work created over the life cycle of a material [29].

Various scenarios can be built in by entering data on the volume of waste managed, broadly categorized by type of material and management practice employed. GHG emissions, electricity saved, and economic effects are computed automatically by WARM based on material-specific parameters for each management practice. Thus, the user may have choices to modify such major factors as landfill gas recovery practices and transport distances to MSW treatment plants. WARM uses a simplified life cycle assessment (LCA) to take impacts into account. In contrast to a complete LCA, involving assessment of health and environmental impacts, the WARM takes account of GHG emissions, carbon stock, and energy effects to give clear and easy-to-implement information on climate change. WARM also simplifies calculation of emissions from lifecycle phases before material disposal, giving an actionable tool for decision-making. WARM bases emission effects on the waste generation origin, as opposed to raw material extraction, since GHG gains are realized by contrast between other waste management options. While it takes into account emissions towards earlier stages, these become relevant only when the compared alternatives involve recycling or source reduction, and even then have effects only on upstream emissions [29].

### 2.2. Plastic Waste Calculation

**Figure 1:**
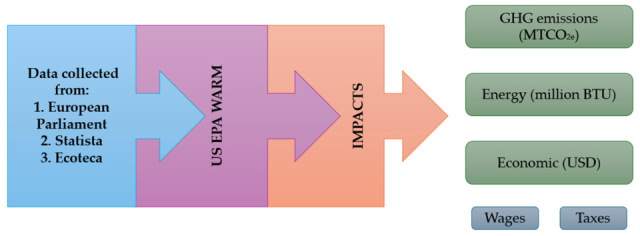
Methodology for determining environmental and economic impacts with the US EPA WARM. Note: Statista is a global online data and business intelligence platform specialized in the collection and visualization of statistical data. Ecoteca is a nongovernmental organization (NGO) from Romania, active since 2010, specialized in waste management, circular economy and climate impact.

To use the US EPA WARM, an inventory of waste quantity data associated with plastic waste management scenarios is required. This involves determining the quantity of waste managed over a specific period, by material type and management method. Accurate data entry ensures reliable comparisons of greenhouse gas emissions, energy savings, and economic and social impacts. Schematically, the methodology used for this paper, with the necessary steps of data collection, modeling, and evaluating impacts, is illustrated in Figure 1.

To ensure the methodological transparency and to address the limitations of available data, especially those related to the Romanian context, certain key assumptions were formulated for the elaboration and interpretation of the US EPA WARM. Table 3 summarizes these assumptions, providing details on their justification and potential impact on the results.

Table 4 presents the data from which the specific scenarios applied in Romania, the European Union (EU), and the United States of America (USA) were derived, providing a comparative perspective on the effects of each option. It is worth mentioning that the data from Romania come from a 2022 study based on plastic packaging waste, and the data for the European Union and the United States come from 2021. Table 5 depicts the PET waste generation and management in the EU and USA.

**Figure 2:**
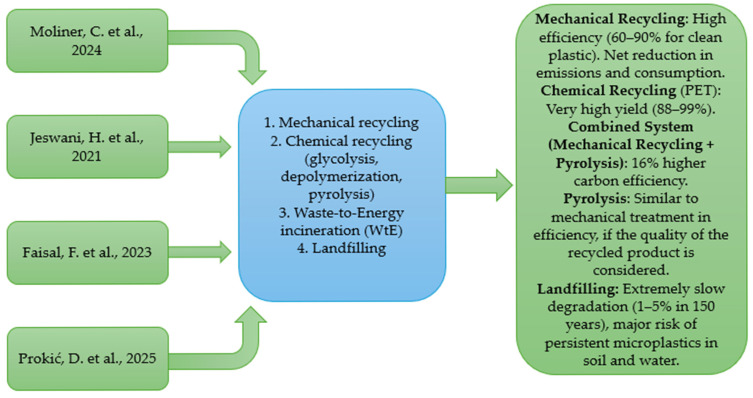
Plastic waste management technologies and key findings from LCA studies Moliner, C. et al. 2024 [35]; Jeswani, H. et al. 2021 [36]; Faisal, F. et al., 2023 [37]; Prokić, D. et al., 2025 [38].

In the specialized literature, there are several LCA studies applied to plastic waste and its end-of-life treatment options. Table 6 highlights several LCA studies related to the main end-of-life treatment options, while Figure 2 depicts a flow diagram for these LCA studies.

## 3. Results

The results obtained highlight the environmental impact of different plastic waste management methods, quantified by using the US EPA Waste Reduction Model (WARM) v16 software. The analysis carried out based on these values allows a detailed assessment of the differences between the three regions in terms of emissions and efficiency of plastic waste treatment.

### 3.1. Carbon Footprint

The carbon footprint was assessed through GHG emissions, expressed in metric kg of carbon dioxide equivalent (Kg CO_2e_). Energy savings were determined through material recovery and reduction in the need to produce new products from primary resources, expressed in millions of British thermal units (BTU) and subsequently converted to gigajoules (MJ).

**Figure 3:**
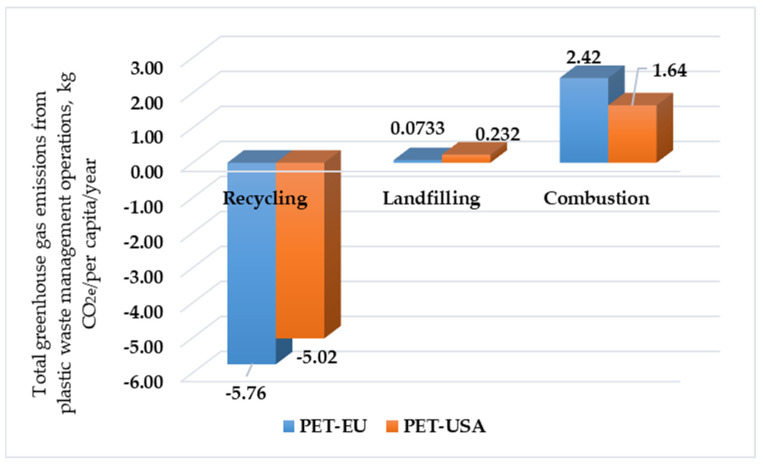
Total GHG emissions for different methods of treating PET in the EU and USA, Kg CO_2e_/per capita/year.

**Figure 4:**
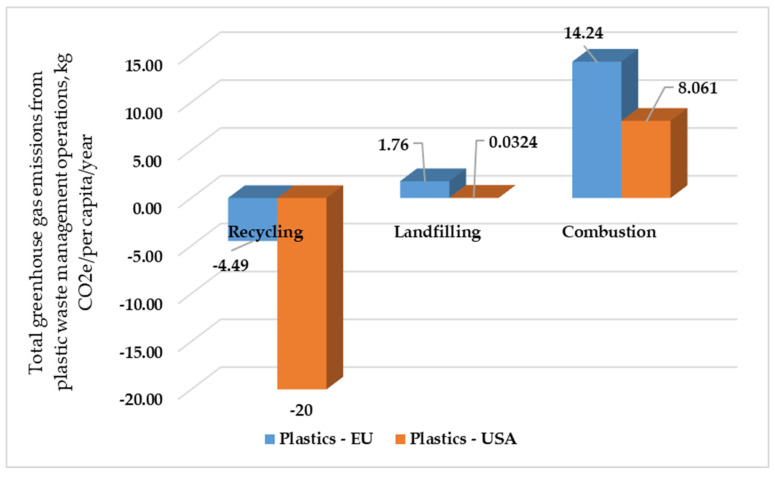
Total GHG emissions for different methods of treating plastic waste in EU and USA, Kg CO_2e_/per capita/year.

Figure 3 presents the total GHG emissions for different processes of treating PET in the European Union and United States of America, while Figure 4 depicts the total GHG emissions for different processes of treating plastic waste in the European Union and United States of America. 

This figure refers to the Kilograms of GHG emissions produced or saved with all PET waste treatment technologies (burning/incineration, landfilling, recycling), using as units Kg CO_2e_/capita/year, for the United States (PET-USA) and the European Union (PET-EU). Both cases are a model of considerable net environmental gain from PET recycling based on negative GHG emissions. This supports the fact that recycling activity stores more emissions than are produced, especially by substituting virgin PET production. The EU had a higher per-capita benefit than the USA, indicating potentially greater effectiveness of recycling plants or processes in the EU. Both regions had net positive PET landfill emissions, which was consistent with the fact that methane (CH_4_) is produced by the biodegradation of organic materials in landfills. US emissions (0.232 Kg CO_2e_/capita/year) were much larger than those of the EU (0.0733 Kg CO_2e_/capita/year). This large disparity suggests that a greater proportion of PET is disposed of at landfills in the US than in the EU. Both regions enjoyed positive net emissions from PET waste incineration/burning. Emissions from burning per capita were greater in the EU than in the US. This could have been due to a greater quantity of PET incinerated per capita in the EU or variation in incineration processes and/or methods of calculating emissions.

The presented bar chart shows the sum of greenhouse gas (GHG) emissions related to different end-of-life management processes of general plastic waste, expressed in Kg of CO_2_ equivalent per capita per year (Kg CO_2e_/capita/year), for both the United States and the European Union. Both economies showed a net environmental gain due to plastic recycling, which was reflected in the negative GHG emission levels. This means that recycling processes (such as collection, sorting, and reprocessing) emit less GHGs than they save, mainly by replacing virgin plastic production. Notably, the United States had a much greater per capita GHG saving through plastic recycling than the European Union. Both had net positive GHG emissions from plastic wastes landfilling, yet the EU per capita landfilling emissions of PET were considerably greater than those for the USA. This contrasts with the earlier observation for PET waste and greater US landfilling emissions. This disparity for overall plastics indicates either a greater percentage of overall general plastic waste being placed in landfills per capita or differences in the efficiency of capture and utilization of landfill gas for total plastic waste. Both areas had positive net emissions of GHGs due to the burning of plastic waste. The USA had considerably lower per capita burning emissions than the EU. This was consistent with possible increased use of incineration as a non-recycled plastic waste end-of-life process of treating waste in the EU or fluctuation in the carbon content of their waste-to-energy plants or accounting standards. The figures differ because of different strategic approaches and environmental profiles caused by plastic waste management in the two nations. While recycling is always a short-term priority GHG reduction method for both, its scale varies. The high per capita rates of landfilling and incineration in the EU, particularly compared with corresponding low rates in the US, are a result of fundamental differences in waste management hierarchies and infrastructures. These differences imply that the EU relies significantly on landfilling and, to a lesser degree, incineration for its nonrecycled plastic waste and that these processes entail higher per capita GHG-related costs for these routes. The reverse, however, indicates the higher per capita benefit of recycling in the US, with comparatively lower emissions related to landfilling and incineration for plastics overall, reflecting different priorities and capacities within the EU and US waste management systems.

### 3.2. Energy Consumption

**Figure 5:**
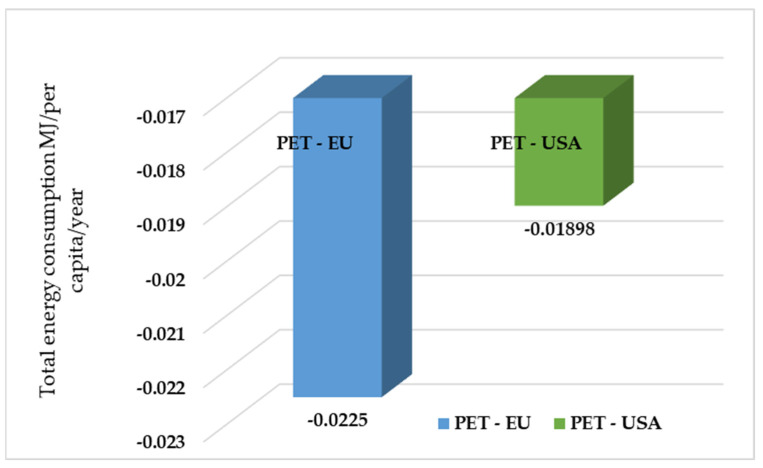
Total energy consumption for different methods of treating PET in EU and USA, MJ/per capita/year.

According to the US EPA WARM, both the European Union and the United States experience negative energy use values. Here, these two figures illustrate the net energy saving per capita (MJ/capita/year) of plastic waste management differentiating the examination of polyethylene terephthalate (PET) and overall plastics, for the United States of America (US) and the European Union (EU). In Figure 4, it can be noticed that EU-Plastics realizes significantly greater energy saving per capita compared to USA-Plastics. The EU energy gain is twice that of the US (−0.0742 vs. −0.0459 MJ/capita/year). This vast disparity indicates that EU has much more efficient plastic management systems and infrastructure per capita compared to the US. And for Figure 5, both regions have a net saving in energy during the PET waste process, which is a positive sign of the contribution by such processes to global energy efficiency. PET-EU records a marginally higher energy saving per capita than PET-USA. This difference, although small in absolute value, suggests a higher energy efficiency of the EU PET waste management system, probably due to higher recycling rates or less energy-intensive PET recycling processes compared to the US. The difference is approximately 0.0283 MJ/capita/year. This reflects the fact that the waste management activities analyzed by WARM provide net energy savings or energy benefits, and not a use in the true sense. By analyzing the magnitude of these benefits, it is observed that the European Union achieves considerably higher energy savings than the United States.

**Figure 6:**
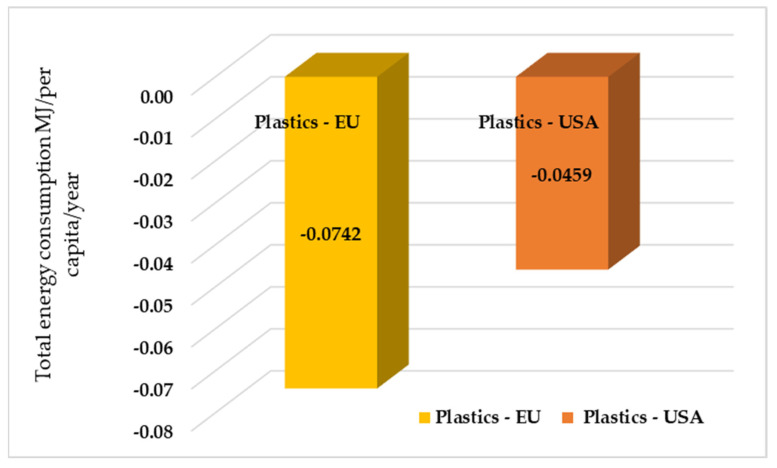
Total energy consumption for different methods of treating plastic waste in EU and USA, MJ/per capita/year.

Figure 6 presents the total energy consumption for different methods of treating plastic waste in EU and USA.

### 3.3. Taxes and Wages

Based on data from the US EPA WARM, total fees associated with plastic waste management differed significantly between the European Union and the United States, reflecting distinct economic approaches and structures. The European Union reported total fees of USD 1,444,353,379.19/year, a considerably higher figure compared with the United States, which reported fees of USD 1,191,900,603.97/year. This difference indicates that the EU collected approximately USD 252.45 million more per year in fees related to plastic waste management than the US. More specifically, fees in the EU were approximately 21.18% higher than those in the US. The European Union reported wage costs of USD 8,792,109,086.4/year, a considerably higher amount than that reported by the United States, USD 4,663,706,674.59/year. This major difference indicates that the EU allocated almost double (to be precise, approximately 1.88 times more) to wages in the plastic waste management sector compared with the US. The combined analysis of environmental and economic impacts reveals divergent approaches between the EU and the US in managing plastic waste. The EU prioritizes investment in labor and the development of recycling value chains (wages indicator), aligning with circular economy objectives. This translates into significant economic benefits linked to job creation. The US demonstrated a substantial climate benefit from recycling but significant positive emissions from incineration. On the economic side, the US appeared to generate a larger tax base from the waste management sector, although this does not necessarily translate into as high a labor intensity in recycling as in the EU. While the EU generated significantly more wages in the PET sector, the US generated more taxes. This may indicate fundamental differences in how economic benefits are distributed and taxed within the waste management chain in the two regions, or in the specific definition of these metrics in the WARM for the EU vs. the US.

### 3.4. Romania in Relation to US EPA WARM Data

Data reached through the US EPA WARM on plastic waste generated in Romania were mixed. Compared with EU and US data, Romanian statistics showed that the country has progressed well towards EU recycling targets and developed infrastructure to support a circular economy. However, structural and operational flaws persist that must be addressed urgently. This in-depth examination of Romania is an insightful case study for the rest of the region’s nations undergoing similar change.

Table 7 presents the data generated by the US EPA WARM for Romania.

From a production standpoint of GHG emissions, 43 kg CO_2e_ per capita is a positive, high value, showing that emissions would be released if virgin material rather than recycled material were used. Recycling itself is a force to be reckoned with in anti-climate-change campaigns, with an added benefit of −18 kg CO_2e_ per capita. This is a negative value, showing that there is a net decrease in GHG emissions, meaning recycling plastic involves much lower energy input and emissions than producing new plastic from virgin material. This way, by recycling, Romania saves 18 kg CO_2_ equivalent emissions per person, clearly demonstrating the immense advantage of this process. Landfilling, conversely, involves the emission of 0.06 kg CO_2e_ per capita, a positive value showing emissions attributed to plastic waste in landfills. Although the direct effect of plastic in landfills may not be profound, it is still a case of contamination and loss of resources. For the energy aspect, the −0.08 MJ/year per capita energy consumption figure represents a net energy saving or energy gain. When interpreted in terms of US EPA WARM and plastic waste management, the figure means that the operations involved here, especially recycling, conserve extra energy (through prevention of manufacture from virgin feedstocks) or even produce energy (through energy recovery). This enormous conservation of energy proves that Romania achieves its actual energy gains from effective plastic waste management. Financially, the management of plastic waste in Romania is extremely expensive. A total of USD 3.90 per capita per year in taxes is likely to include landfill taxes, environmental taxes, or payments into Extended Producer Responsibility (EPR) schemes. Such an expenditure signals an attempt to avoid landfilling and promote recycling or other environmentally friendly means of disposing of waste. In addition to taxation, the sector also incurs the cost of labor, worth USD 24.02 per capita per year. This number indicates the significant labor force that goes into this sector, be it in collection and sorting, processing, and administration, thus underlining the socioeconomic contribution of the waste management sector through employment and livelihoods.

## 4. Discussion

Plastic waste management analysis is much more than a matter of rough comparative statistics and involves setting the results arrived at with the US EPA WARM in the context of world sustainability, economic growth, and climate crisis. Throughout the world, plastic production and consumption rose exponentially into a trash crisis of unparalleled environmental consequence. The effects are felt through ocean and land ecosystem contamination, microplastics in the food system, and high levels of GHG emissions throughout the entire life cycle of plastic. Proper plastic waste management in this scenario is not only an environmental issue but an important economic and social issue that affects public health, economic prosperity, and the ability of the planet to support life. The European Union, the United States, and Romania are compared here based on US EPA WARM data to show that they essentially have different political and investment strategies for this problem. The European Union, with its quintessential focus on the circular economy, ambitious recycling rates, and producer responsibility policies, proves that stringent regulation and strong investment in recycling plants (as reflected in higher wages and taxation) can translate into real benefits in terms of energy and GHG emission reduction. The model presents the trajectory towards the green future, rendering wastage an asset instead of a loss. In contrast, the more hands-off and frequently disjunctive American strategy, effective as it is at processing enormous amounts of rubbish, is more likely to rely on high-environmental-impact solutions such as landfilling and combustion. This is reflective of deep disparities in economic and environmental priorities, with the short-term efficiency of refuse management in the American case too frequently trumping longer-term aims of carbon footprint reduction and resource conservation. Romania, as an EU member state, is in transition and alignment, and it has traditional challenges of a historical nature around infrastructure and mindset. However, it is taking significant steps, for example, introducing the deposit-return system (DRS), to demonstrate its capacity to significantly improve the collection and recycling of certain forms of plastic packaging, such as PET. Its own performance, though smaller in absolute terms than that of the EU and the US, is critical to the European environment and can offer the potential for quick improvement with the adoption and refinement of best practice.

For a deeper analysis, further investigations are needed in several key areas: (1) longitudinal examination of the evolution of policy impacts to identify optimal timing of fiscal instruments; (2) disaggregated analysis of Member States to highlight good practice models and adapt strategies to regional specificities; (3) integration of social cost accounting to capture broader economic externalities; (4) development of hybrid LCA–economic models, specifically calibrated for emerging economies, to optimize waste management processes; and (5) investigation of the influence of behavioral economics factors on recycling participation rates, depending on different socioeconomic contexts. These research directions can significantly contribute to improving the effectiveness of environmental policies and promoting more sustainable waste management practices.

### Practical and Policy Implications

While the European Union has adopted a proactive and regulated approach focused on recycling and the circular economy for managing plastic waste, the United States still relies more heavily on landfilling and exports. These distinct approaches are the result of diverse economic and policy factors, which are highlighted in Table 8 and Table 9.

**Figure 7:**
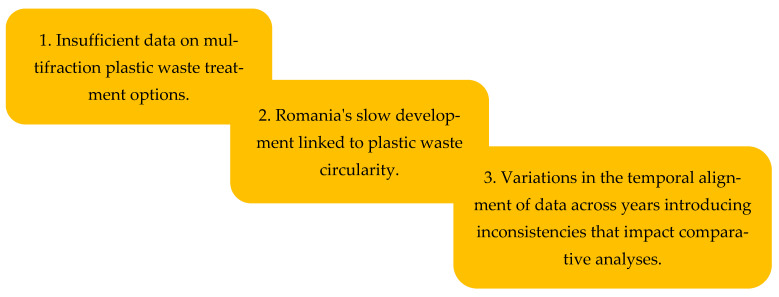
Shortages in research referring to data gathered from Romania, the EU, and the USA.

Figure 7 depicts the main shortages in research referring to data gathered from Romania, the European Union, and the United States of America.

## 5. Conclusions

This study has provided a comparative assessment of end-of-life plastic waste management strategies in the European Union and United States of America, also integrating an analysis of the Romanian context and progress towards the circular economy. The analysis validated the initial premise that variations in policy regimes and levels of investment in infrastructure lead to distinct, measurable effects, both environmentally and economically. Although the scope of the research was limited by the data aggregation and specific parameters of the US EPA WARM v16 model, the results obtained contributed to the identification of sustainable solutions.

Key messages from this analysis:

1. The importance of recycling. The analysis demonstrated that recycling is the most effective plastic waste management strategy for reducing GHG emissions. The significant benefits in avoided emissions, particularly those for general plastic in the USA (−20 kg CO_2e_/capita/year) and for PET in the EU (−0.0225 MJ/capita/year energy savings), underline its essential role in the transition to a low-carbon economy.
2. Regional differences and opportunities for improvement. There are clear differences in the carbon footprint and energy efficiency of waste management between the EU and the US. Although the EU achieves higher per capita energy savings for total plastic, it faces considerably higher per capita emissions from incineration and landfilling. In contrast, the US, with a remarkable benefit from recycling, has lower emissions from incineration and landfilling for overall plastic, but an untapped potential in systematically scaling up energy savings at the total plastic level compared with the EU. For Romania, positioning itself within the European dynamics offers both challenges and opportunities to learn from best practices at EU level and to accelerate its progress towards circular economy goals, by increasing recycling capacity and reducing reliance on landfilling.
3. Economic implications: The study highlights that waste management policies have distinct economic implications. The EU demonstrates much higher wage generation in the PET sector (over $2.8 billion), indicating a more labor-intensive infrastructure and a deep commitment to the circular economy. In contrast, the US generates a larger tax base (over $2 billion), suggesting different tax structures or volumes of economic activity.
4. The need for a multifaceted and integrated approach: Beyond technological solutions, long-term success depends on a systemic change in social behavior supported by education and public awareness campaigns. An inclusive approach is imperative, stimulating positive collaboration between policymakers, businesses, research centers, and civil society organizations. Romania, in this context, can play an active role in promoting increased public awareness and environmentally friendly consumption and waste management practices, contributing to a cleaner and more resource-efficient future.

In summary, the analysis reconfirms that active recycling and its expansion are central pillars for reducing the climate and energy impact of plastic waste. Navigating effectively towards a circular economy requires recognizing and adapting to regional dynamics, implementing evidence-based policies, and promoting a culture of sustainability through collective efforts at national and global scales, and how developing countries should strengthen a strong strategy for targeting circularity.

## Figures and Tables

**Table 1:** Main polymer categories and their codes.

Code	Polymer Type	Examples	Degree of Recycling	References
1	PET (polyethylene terephthalate)	Beverage bottles, packaging	Easily recyclable	[2,6]
2	HDPE (high-density polyethylene)	Milk cans, detergents	Easily recyclable	[7,8]
3	PVC (polyvinyl chloride)	Pipes, windows, packaging	Difficult; requires dechlorination, chemical recycling	[9]
4	LDPE (low-density polyethylene)	Bags, foils	Limited recycling, sometimes mechanical	[10]
5	PP (polypropylene)	Containers	Mechanical or chemical recycling, variable acceptance	[11]
6	PS (polystyrene)	Casseroles, glasses, packaging	Difficult, sometimes chemical recycling	[12]
7	Other types (e.g., polycarbonate, bioplastics)	CDs, special bottles	Rarely recycled, special treatments	[7]

**Table 2:** Advantages of individual polymer analysis vs. mixed fractions.

Polymer Appearance	Individual Analysis	Mixed Fraction	References
Composition identification	Precise	Limited/uncertain	[14,15]
Material quality	High	Variable/low	[14,15]
Recycling efficiency	Optimized	Low	[14,15]
Processing costs	Potentially smaller	Often larger	[14,15]

**Table 3:** Key assumptions for US EPA WARM modeling.

No.	Parameters	Applied Hypothesis	Justifications and Sources	Impact on Results
1	Data on plastic waste combustion from Romania	No specific data were included for the combustion of plastic waste in Romania.	Relevant and validated data on the amount of plastic waste incinerated in Romania were not available for the study year. Therefore, this stream was excluded from the LCA analysis for Romania.	Potentially underestimates the energy impact and emissions associated with incineration in Romania, if it occurs on a significant scale.
2	Source of data on plastic waste (Romania)	Data on plastic waste quantities were taken from Ecoteca.	Ecoteca is a recognized source of data and reports on waste management in Romania. [Source: https://ecoteca.ro/evolutia-ambajelor-puse-pe-piata-si-valorificate-in-perioada-2009-2022-in-romania.html (accessed on 11 June 2025)]	Ensures the use of data specific to the Romanian context.
3	Consistency of data sources across regions (EU vs. US)	Data for the EU were taken directly from the European Parliament, while for the US, aggregated data from the Statista platform were used.	This approach was necessary because of the accessibility and availability of data.	Potential variations in the collection methodologies and definition of indicators between Eurostat’s primary sources and those used by Statista for the US could influence the accuracy of direct comparisons between the two entities.
4	Year of data (Romania vs. US/EU)	Data from 2022 were used for Romania, while data from 2021 were used for the US and EU.	Complete and consolidated data for plastic waste in Romania for 2021 were not available at the time of collection; 2022 was the most recent year with complete data. It is assumed that the major trends and structure of waste streams did not change radically between 2021 and 2022 to affect overall comparability.	This may introduce a slight temporal discrepancy in direct comparisons, but it is a necessary approach in the absence of uniform data.
5	Data normalization (all regions)	All numerical data were reported per capita for each region.	This normalization ensures a fair and scientifically valid comparison between regions with different populations, eliminating the influence of absolute population size.	It allows for a more relevant interpretation of waste management performance at the individual/societal level.
6	Converting percentages to absolute volumes (US and EU)	The estimated percentages for each waste stream (recycling, incineration, landfill) were converted into absolute quantities (tons) using the total volume of PET waste generated. For percentage ranges (e.g., 25–30%), the average value (e.g., 27.5%) was used.	This conversion was necessary to feed the WARM with absolute numerical data and to ensure a coherent representation of material flows. The use of the average is standard practice in the absence of a clear indication for another value.	Absolute and per capita results depend on the accuracy of the percentages estimated in the original sources. The use of the average introduces an assumption that could slightly over/underestimate the actual volumes for each treatment category.
7	Composition of plastic waste (Romania)	A composition similar to the EU average for mixed plastic was assumed, in the absence of detailed data by polymer type (PET, HDPE, etc.) from the Ecoteca source.	Detailed data by polymer type for the total plastic waste stream in Romania were not available at the level required for modeling. This assumption was necessary to align the data with the WARM categories.	This could have affected the accuracy of the LCA assessment for individual polymers, as the WARM has different factors for each polymer type.
8	Average waste transport distance (Romania)	An average distance of 100 km was estimated for transporting waste to treatment/disposal facilities.	This value is an estimate based on average transport distances in European countries with similar population density and waste management infrastructure. There is no centralized, official data.	An underestimation/overestimation of distance could slightly influence transportation emissions and fuel consumption in the WARM.
9	Energy efficiency of mechanical recycling (Romania)	WARM default values were applied for the energy efficiency of mechanical recycling processes.	No specific data on the energy efficiency of recycling facilities in Romania were available. WARM values are considered a reasonable starting point in the absence of local data.	The values used may differ from the actual performance of Romanian installations, affecting the calculation of energy savings.

**Table 4:** Kilograms of plastic waste from different countries.

Romania				Reference
Kilograms generated per capita	Kilograms recycled per capita	Kilograms landfilled per capita	Kilograms combusted per capita	[30]
25.7	22.3	3.3	-	
**European Union**				**Reference**
Kilograms generated per capita	Kilograms recycled per capita	Kilograms landfilled per capita	Kilograms combusted per capita	[31]
24.7	18	1.3	5.3	
**United States of America**				**Reference**
Kilograms generated per capita	Kilograms recycled per capita	Kilograms landfilled per capita	Kilograms combusted per capita	[32]
≈150	≈7.1	≈126.4	≈16.5	

Note: Romania—19.06 million inhabitants; EU—449.2 million inhabitants; USA—340.1 million inhabitants.

**Table 5:** PET waste generation and management in the EU and USA.

Region	Category Waste—PET	Total Quantity (Tons)	Percentage of Total Generated (%)	Quantity per Capita (Kg/Capita)	References
EU	Total generated	5,000,000	100%	≈11	[31]
	Recycled	2,500,000	50%	≈5	
	Combusted	875,000	30–40%—35% calculation	≈1	
	Landfilled	1,625,000	10–20%—15% calculation	≈3	
USA	Total generated	6,000,000	100%	≈17	[33,34]
	Recycled	1,650,000	25–30%—27.5% calculation	≈4	
	Combusted	450,000	5–10%—7.5% calculation	≈1	
	Landfilled	3,900,000	60–70%—65% calculation	≈11	

**Table 6:** Key paper for plastic waste end-of-life treatment technologies considering LCA studies.

Articles/References	Technology Focus	Energy Consumption	GHG Emissions	Yield Efficiency/Key Results
“Municipal Plastic Waste Recycling through Pyrogasification” [35]	Mechanical recycling, chemical recycling (glycolysis, depolymerization)	Mechanical: 0.5–2.5 MJ/kg; chemical: 1.5–10 MJ/kg	7–88% less than alternatives	Mechanical: 60–90% (clean); chemical: 88–99% (PET)
“Life Cycle Environmental Impacts of Chemical Recycling via Pyrolysis of Mixed Plastic Waste in Comparison with Mechanical Recycling and Energy Recovery” [36]	Mechanical recycling, pyrolysis, combined	Mechanical: 13.32 MJ/kg; pyrolysis: higher	0.48 kg CO_2e_/kg (combined)	Combined: 16% higher carbon efficiency
“Pyrolytic Conversion of Waste Plastics to Energy Products: A Review on Yields, Properties, and Production Costs” [37]	Pyrolysis vs. mechanical recycling, waste-to-energy incineration (WtE)	Pyrolysis: ~50% lower than WtE	Pyrolysis: −0.45 t CO_2e_/t; WtE: 1.89 t CO_2e_/t	Similar to mechanical treatment if recycled quality is considered
“Evaluating Plastic Waste Management in EU Accession Countries: A Life Cycle Perspective from the Republic of Serbia with Microplastic Implications”, [38]	Landfilling, mechanical recycling, incineration	Landfilling: ~580 MJ/ton PET; mechanical: ~1.450 MJ/ton PET; incineration: ~2.100 MJ/ton PET	Landfilling: ~1.280 kg CO_2e_/t; mechanical: ~530 kg CO_2e_/t; incineration: ~870 kg CO_2e_/t	Landfilling: Slow degradation—only 1–5% in 150 years. Major risk of persistent microplastics in soil and water; mechanical: avoided emissions and consumption (LCA net benefits); incineration: recovered electricity. Toxic emissions: dioxins, furans. Microplastics detected in ash and slag

**Table 7:** Data generated by US EPA WARM for Romania.

**Romania**	**GHG emissions from production** (Kg CO_2e_)/per capita/year	**GHG emissions from recycling** (Kg CO_2e_)/per capita/year	**GHG emissions from landfilling** (Kg CO_2e_)/per capita/year
	≈43	−18	≈0.06
	Energy Consumption MJ/per capita/year		
	≈−0.08		
	Taxes $/per capita/year		
	≈3.90		
	Wages $/per capita/year		
	≈24.02		

**Table 8:** Economic and policy factors in plastic waste management for the countries studied.

Main Feature	United States of America	European Union	Romania	References
Landfill/incineration costs	Lower ($55/ton). Reduces economic incentives for recycling.	Higher ($80–125/ton). Make recycling more economically attractive.	Lower costs but rising because of EU pressure.	[39,40]
Regulatory targets	No federal recycling mandates; state-level variation.	Ambitious recycling quotas (50% by 2025, 55% by 2030); strict directives.	Bound by EU targets but struggles with implementation.	[39,40]
Producer responsibility (PR)	Limited or nonexistent PR policies at the national level. Producers are less accountable.	Extensive and mandatory REP schemes. Producers are responsible for the waste management of their products.	Extensive and mandatory REP schemes. Producers are responsible for the waste management of their products.	[39,40]
Circular economy objectives	Less developed. Focus on exporting low-value plastics, not developing domestic markets.	Strategic priority. Promoting closed-loop recycling, secondary materials markets, design for recycling.	Dependent on national capacity to enforce EU rules.	[39,40]

**Table 9:** Practical and policy implications of the plastic waste management study.

Central Theme	Practical Implications	Policy Implications
1. Reconfirming recycling priority and expanding capacity	Plastic and packaging companies should intensify efforts in design for recycling and use of recycled content. Private investment should be stimulated in modern sorting and recycling facilities (mechanical, chemical). Consumers should be continuously educated on the importance and correct methods of sorting at source.	Mandatory recycling targets: Expansion and strict enforcement for various types of plastic. Extended Producer Responsibility (EPR): Strengthening and expanding EPR schemes for financial accountability of producers throughout the entire life cycle (collection, sorting, recycling). Recycled content standards: Minimum requirements for new products and packaging, creating a stable market for recycled materials.
2. Reducing dependence on positive-emissions methods (landfilling and incineration)	Sanitation operators and municipalities should prioritize diversifying treatment options, reducing reliance on landfill and direct incineration in favor of recycling. Advanced energy recovery technologies (if needed for nonrecyclables) should be optimized to minimize residual emissions.	Landfill and incineration taxes: Imposing or increasing these taxes to discourage the practices and direct flows towards recycling. Progressive bans: Implementing bans on the landfilling of certain types of recyclable plastic waste.
3. Romania’s role in the European context	The implementation of European directives on plastic waste management should be accelerated, with focus on developing selective collection and recycling capacities, especially in rural and poorly covered urban areas. Infrastructure should be modernized to reduce dependence on landfill.	Romanian authorities should develop and implement specific national policies to integrate EU recycling objectives, stimulate investments in clean technologies, and implement economic instruments (e.g., landfill taxes). Public education and awareness campaigns should mobilize the population in correct sorting.

## Data Availability

Data mentioned in this study is available from the first author.

## Abbreviations

The following abbreviations are used in this manuscript:

HDPE	High-density polyethylene
LDPE	Low-density polyethylene
PP	Polypropylene
PE	Polyethylene
PVC	Polyvinyl chloride
PS	Polystyrene
NIR	Near-infrared spectroscopy
MPW	Mismanaged plastic waste
DRS	Deposit-return system
AI	Artificial intelligence
EU	European Union
US	United States of America
EPR	Extended Producer Responsibility
